# Impact of Parkinson's Disease on Caregiver Quality of Life in Japan

**DOI:** 10.1002/mdc3.13700

**Published:** 2023-03-14

**Authors:** Koichi Nagaki, Ryoko Nakagawa, Miwako Ishido, Yoko Yoshinaga, Jun Watanabe, Kanako Kurihara, Yuka Hayashi, Hiromu Ogura, Takayasu Mishima, Shinsuke Fujioka, Yoshio Tsuboi

**Affiliations:** ^1^ Department of Neurology, Faculty of Medicine Fukuoka University Fukuoka Japan; ^2^ Medical, AbbVie GK Tokyo Japan

**Keywords:** Parkinson's disease, caregivers, PDQ‐Carer, quality of life, Japan

## Abstract

**Background:**

Parkinson's disease (PD) adversely affects the quality of life (QoL) of not only patients but also their caregivers.

**Objective:**

To determine the factors that most impact the QoL of family caregivers of patients with PD in a large Japanese population using data from the Japanese Quality‐of‐Life Survey of Parkinson's Disease (JAQPAD) study.

**Methods:**

Questionnaires, including the Parkinson's Disease Questionnaire‐Carer (PDQ‐Carer), were distributed to patients and their caregivers. Univariate and multivariate regression analyses were performed with the PDQ‐Carer Summary Index (SI) score as the dependent variable to determine the factors that impact caregiver QoL.

**Results:**

Overall, 1,346 caregivers were included in the analysis. Female sex, unemployment, caring for a patient with a high‐level need for nursing care, and a high Nonmotor Symptoms Questionnaire score were factors with a significant negative impact on caregiver QoL.

**Conclusion:**

Results from this study identified several factors that affect caregiver QoL in Japan.

Parkinson's disease (PD) is a complex, heterogeneous neurodegenerative disorder characterized by progressive motor symptoms, including tremor, rigidity, and bradykinesia, and many nonmotor symptoms, such as cognitive impairment, neuropsychiatric symptoms, autonomic dysfunction, pain, and fatigue.^1^ With improvements in diagnostic accuracy, the availability of medications, such as levodopa, for symptom control, and the longer life expectancy of patients with PD, many patients and their families now live with the burden of the disease for a considerable period of time.^2^, ^3^, ^4^ Although l‐dopa is still regarded as the most effective medication for the symptomatic treatment of PD, long‐term treatment with l‐dopa is often associated with a range of motor complications, including wearing‐off (WO), dyskinesia, and complex fluctuations in the advanced stage of the disease.^5^ Consequently, PD imposes a significant burden on patients as well as their families and caregivers.^6^, ^7^

Diverse symptoms of PD affect the quality of life (QoL) of patients and their caregivers from various perspectives. Therefore, factors associated with a negative impact and the ways to improve the QoL of patients with PD and their caregivers have been highlighted.^8^ Although factors affecting caregivers' QoL are recently being identified, only a few studies have examined all factors together, including PD symptoms, patient characteristics, and caregiver characteristics that affect the caregivers’ QoL.

The Japanese Quality‐of‐Life Survey of Parkinson's Disease (JAQPAD) study was undertaken to assess the impact of PD on the QoL of both patients and their caregivers in Japanese real‐world clinical practice.^9^ Results from the QoL analysis of patients showed that “off time” and severity of nonmotor symptoms were the major determinants of QoL in Japanese patients with PD. Herein, we present the results for caregiver QoL in PD to enhance the current understanding of the factors that most impact caregiver QoL within the dynamics of the Japanese family structure.

## Methods

### Study Design and Methods

Full details of the study design and methods have been reported previously.^9^ Briefly, this was an observational, cross‐sectional study conducted in Japan between August 14, 2017, and October 31, 2017. The study was conducted in accordance with the Declaration of Helsinki (1964), was compliant with the local laws and regulations, and was performed in accordance with the guidelines for Good Pharmacoepidemiology Practices in noninterventional studies and the Japan Ethical Guidelines for Medical and Health Research Involving Human Subjects.^10^

### Eligibility

All 8,183 members of the Japan Parkinson's Disease Association (JPDA) were invited to participate in this study. Caregivers who were currently caring for adult patients with a confirmed diagnosis of idiopathic PD and who were willing and able to provide informed consent were included in the study. Caregivers of patients with missing or disqualifying medical information (unavailability of information on medication or the frequency of medication, taking ≥14 different types of medication, or taking medication ≥9 times a day) were excluded from the study as they were considered to have deviations from the maximum possible combination of the prescription for PD in Japan. Caregivers who were not family members and caregivers of patients who did not provide informed consent were also excluded.

### Study Outcomes and Assessments

The primary outcome of this study was the impact of PD on caregiver QoL, as assessed by the Parkinson's Disease Questionnaire‐Carer (PDQ‐Carer) Summary Index (SI) score.^11^ The nonmotor symptoms of PD were assessed using the Nonmotor Symptoms Questionnaire (NMSQ).^12^ Please see Data S1: Supplementary Text for details about QoL and activities of daily living (ADL) measures used for study outcomes and assessments. Permissions were obtained for the use of the PDQ‐Carer (Oxford University) and NMSQ (International Parkinson and Movement Disorder Society).

### Statistical Analyses

Continuous and categorical variables are presented as mean [±standard deviation (SD)] and number (%), respectively. Univariate analysis to test the significance of the regression coefficient using *t*‐distribution was performed to identify predictor variables with the strongest association with caregiver QoL. Multiple regression analyses using generalized linear models were conducted using PDQ‐Carer SI as the main outcome measure, with independent variables determined as described in Data S1: Supplementary Text. We selected some individual variables to include factors with a high correlation with the objective variable (PDQ‐Carer SI) and at the same time prevent multicollinearity between factors. Finally, “off time” duration (forced‐in covariate), sex and working status of caregiver, nursing care level of the patient, frequency and type of oral PD medications taken daily, duration of troublesome dyskinesias, the 9‐item Wearing‐Off Questionnaire (WOQ‐9) score, and NMSQ total score (TS) were considered independent variables. The backward elimination method was used for variable selection in the models.

## Results

### Patient Demographics and Baseline Characteristics

As shown in Figure S1, a final dataset of 1,346 caregivers was used in the study. Table 1 shows the sociodemographic characteristics of the caregivers. The baseline demographics and clinical characteristics of patients are shown in Table S1.

**TABLE 1:** Sociodemographic characteristics of caregivers

Caregiver demographics	N = 1,346
Age (yr), mean (SD) (n = 1,340)	69.6 (9.5)
<65, n (%)	298 (22.1)
≥65, n (%)	1,042 (77.4)
Sex	
Female, n (%)	736 (54.7)
Period of care (yr), mean (SD) (n = 1,293)	6.6 (5.4)
Time spent on caregiving, hr/week, mean (SD) (n = 1,287)	66.6 (49.8)
Relationship with the patient, n (%)	
Spouse	1,215 (90.3)
Child	104 (7.7)
Parent	17 (1.3)
Sibling	13 (1.0)
Relative other than above	8 (0.6)
Live‐in caregiver, n (%)	1,346 (100.0)
Working status, n (%)	
Full‐time worker	135 (10.0)
Part‐time worker	146 (10.8)
Other (eg on leave from work)	49 (3.6)
Student	1 (0.1)
Housewife/stay‐at‐home husband	490 (36.4)
Seeking a job/unemployed	17 (1.3)
Retired	398 (29.6)
Other	101 (7.5)
Nursing care level of the patient,^a^ n (%)	
None	145 (10.8)
Assistance 1	98 (7.3)
Assistance 2	215 (16.0)
Care level 1	146 (10.8)
Care level 2	320 (23.8)
Care level 3	192 (14.3)
Care level 4	100 (7.4)
Care level 5	61 (4.5)
Unknown	69 (5.1)

^a^ There are seven levels of long‐term care need certificates in Japan.

Abbreviation: SD, standard deviation.

### Caregiver QoL, as Assessed by the PDQ‐Carer

Overall, the mean PDQ‐Carer SI score was 39.3 (Table S2). The PDQ‐Carer scores by Hoehn Yahr (H&Y) stages of the patients are shown in Table S3.

### Factors Impacting Caregiver QoL

The univariate analysis demonstrated that duration of “off time,” patient nursing care level, NMSQ TS, sex, working status of the caregiver, types of oral PD medications taken daily, frequency of oral PD medications a day, and duration of troublesome dyskinesia were individually predictive of changes in the 8‐item Parkinson's Disease Questionnaire (PDQ‐8) SI score (Table 2).

**TABLE 2:** Multivariate regression analyses of the Parkinson's Disease Questionnaire‐Carer (PDQ‐Carer) scores

Variable	Univariate model			Multivariate initial model					Multivariate final model backward elimination *P* < 0.05				
	CE	95% CI	*P*‐value	CE	Standardized CE	VIF	95% CI	*P*‐value	CE	Standardized CE	VIF	95% CI	*P*‐value
Duration of “off time”	0.855	0.263, 1.447	0.0047	0.033	0.0035	1.1029	−0.560, 0.626	0.9131	−0.039	−0.0041	1.0361	−0.602, 0.525	0.8931
Patient nursing care level	6.179	5.539, 6.820	<0.0001	5.715	0.4681	1.1676	4.921, 6.508	<0.0001	5.476	0.4494	1.1143	4.722, 6.231	<0.0001
NMSQ total score	1.534	1.258, 1.811	<0.0001	0.885	0.1998	1.1463	0.600, 1.170	<0.0001	0.893	0.2028	1.0918	0.623, 1.163	<0.0001
Sex (ref: female)	8.498	−11.291, −5.706	<0.0001	−5.312	−0.1112	1.0270	−8.223, −2.400	0.0004	−5.506	−0.1155	1.0093	−8.315, −2.697	<0.0001
Working status of the caregiver (ref: unemployed)	9.346	−12.544, −6.147	<0.0001	−4.436	−0.0827	1.0352	−7.719, −1.153	0.0082	−4.815	−0.0895	1.0234	−8.006, −1.624	0.0032
Types of oral PD medications taken daily	0.984	0.434, 1.534	0.0005	−0.338	−0.0355	1.1232	−0.945, 0.269	0.2745					
Frequency of oral PD medications a day	0.899	0.016, 1.783	0.0461	−0.507	−0.0339	1.1824	−1.485, 0.472	0.3098					
Duration of troublesome dyskinesia	1.193	0.260, 2.125	0.0122	0.123	0.0081	1.0975	−0.839, 1.086	0.8012					
WOQ‐9 (ref: absence)	1.209	−1.839, 4.256	0.4366	−0.002	0.0000	1.0993	−3.256, 3.252	0.9988					

Abbreviations: CE, coefficient estimate; CI, confidence interval; NMSQ, Nonmotor Symptoms Questionnaire; PD, Parkinson's disease; ref, reference; VIF, variance inflation factor; WOQ‐9, 9‐item Wearing‐Off Questionnaire.

Results from the multivariate regression analyses showed that female sex, caregiver unemployment, worse nonmotor symptoms, and higher level of nursing care were associated with a worse caregiver QoL (Table 2). Patient nursing care level and NMSQ TS, especially, showed high scores for standardized coefficient estimate, indicating that these two predictors had a higher impact on the caregivers’ QoL score. Interestingly, patient “off time” did not significantly affect caregiver QoL.

### Relationship between PDQ‐Carer Scores and NMSQ Subdomain Scores

The most commonly reported nonmotor symptoms were urinary (mean NMSQ score, 1.6 of 2 questions) and memory (2.1 of 3 questions) dysfunctions (Table S4).

The PDQ‐Carer SI score and subdomain scores correlated significantly with all NMSQ subdomain scores (Table S5). Of the nine NMSQ subdomains, perceptual correlated most strongly with the PDQ‐Carer SI score, followed by memory, sleep disorders, and digestive disorders.

## Discussion

This observational, cross‐sectional study conducted in Japan assessed the impact of PD on the QoL of family caregivers of the patients.

In this study, the mean PDQ‐Carer SI score was 39.3, suggesting that the average QoL of caregivers was not severely compromised. Similar results were seen in a European multi‐country cross‐sectional study.^13^ Moreover, even caregivers of patients receiving device‐aided therapy (DAT) were reported to have only mild to moderate burden.^14^ A possible explanation for this result could be that the improvements in caregiver QoL due to improved PD symptoms outweigh the hassle of DAT. Our observation that caregivers of patients with severe disease (H&Y stages 4 and 5) had worse PDQ‐Carer scores (Table S3) supports this hypothesis.

Results from the multiple regression analyses revealed that female sex, unemployment, caring for a patient with a high‐level need for nursing care, and a high NMSQ score were factors with a significant negative impact on caregiver QoL (Table 2).

Previous reports from the literature on the impact of sex of the caregiver on their QoL have been somewhat conflicting.^15^, ^16^, ^17^ Therefore, while sex of the caregiver may influence caregiver QoL, its impact on caregiver burden may be country specific and dependent on different family dynamics and structures around the world. In line with results from other studies on PD, as well as other chronic disease states,^18^, ^19^, ^20^ better QoL was observed among caregivers who were employed, possibly as employed caregivers generally have a wider social network and therefore interact with a range of people, thereby reducing their emotional distress.

Interestingly, while we previously reported that “off time” duration and severity of nonmotor symptoms adversely affected patient QoL,^9^ “off time” duration was not significantly associated with the PDQ‐Carer SI score. A possible explanation for this may be that while “off time” is frequently reported by patients and accompanied by a reduction in patient QoL,^21^ nonmotor symptoms of PD, such as pain, depression, and sleep disturbances, more directly affect the well‐being of the caregiver.^22^, ^23^ In this study, among NMSQ subdomains, perceptual, memory, and sleep disorders showed moderate correlation with the PDQ‐Carer SI score (Table S5). Surprisingly, mood showed weak correlation with the PDQ‐Carer SI score, which is in contrast to previous reports that showed that patient mood impacts caregiver burden.^6^, ^24^ This result suggests that perceptual and memory dysfunction, including dementia, related to higher H&Y stages may strongly affect caregiver QoL in the Japanese population.

The following limitations of this study should be acknowledged. First, the study was limited to patients and caregivers willing to complete a survey, imparting a selection bias; therefore, the data may not be fully generalizable. Second, although self‐reporting of data was considered the most appropriate method to meet the study objective and the questionnaires used have also been validated in previous studies, the limitations associated with self‐reported data must be considered. Third, use of the JPDA database may have contributed to a negatively skewed distribution of patients in terms of symptom severity; consequently, the sample population may not be fully representative of the Japanese population. Fourth, only disease‐related factors were analyzed in this study. The lack of information on care service usage and the socioeconomic status of patients and caregivers must be acknowledged. Fifth, the lack of information regarding severity of the motor symptoms and cognitive impairment should be acknowledged. Lastly, this was a cross‐sectional study, and therefore further longitudinal studies are required to establish a link between lesser‐known caregiver factors and QoL and to provide greater clarity on caregiver burden over time. However, a key strength of this study is its large sample size, which is considerably greater than that of previously reported studies.^23^, ^24^, ^25^, ^26^ Furthermore, this is one of the few observational studies of its kind that enabled an extensive assessment of the impact of PD on caregiver QoL in Japan.

To summarize, nonmotor symptoms are often unrecognized and underestimated when planning therapeutic strategies in a clinical setting. Results from this subanalysis of the JAQPAD study underscore the importance of recognition and management of nonmotor symptoms, which significantly affect caregiver QoL.

## Author Roles

(1) Research Project: A. Conception, B. Organization, C. Execution; (2) Statistical Analysis: A. Design, B. Execution, C. Review and Critique; (3) Manuscript Preparation: A. Writing of the first draft, B. Review and Critique.

K.N.: 1C, 2C, 3B.

R.N.: 1A, 1C, 2B, 2C, 3B.

M.I.: 1A, 1B, 1C, 2B, 2C, 3A.

Y.Y.: 2A, 2B, 2C, 3B.

J.W.: 1C, 2B, 2C, 3A.

K.K.: 1C, 2C, 3B.

Y.H.: 1C, 2C, 3B.

H.O.: 1C, 2C, 3B.

T.M.: 1C, 2C, 3B.

S.F.: 1C, 2C, 3B.

Y.T.: 1A, 1B, 1C, 2C, 3B.

## Disclosures

**Ethical Compliance Statement:** The study was conducted in accordance with the Declaration of Helsinki (1964), was compliant with the local laws and regulations, and was performed in accordance with the guidelines for Good Pharmacoepidemiology Practices in noninterventional studies and the Japan Ethical Guidelines for Medical and Health Research Involving Human Subjects.^10^ The JAQPAD study was approved by the external institutional review board of the nonprofit organization MINS (Tokyo, Japan. Approval #170517). An informed consent form was sent to patients and their caregivers by physical mail, and the completed forms were collected anonymously by return mail directly from patients and caregivers to a contracted clinical research organization. We confirm that we have read the Journal's position on issues involved in ethical publication and affirm that this work is consistent with those guidelines.

**Funding Sources and Conflicts of Interest:** This work was supported by AbbVie GK. AbbVie participated in the study design; research; data collection, analysis, and interpretation; and writing, reviewing, and approving the publication. Y.T. has served as an advisor for AbbVie GK. R.N. and J.W. are employees of AbbVie GK and may receive stock or stock options. M.I. and Y.Y. are former employees of AbbVie GK. K.N., K.K., Y.H., H.O., T.M., and S.F. report no conflicts of interest.

**Financial Disclosures for the Previous 12 Months:** Y.T. has received research support from the Japan Agency for Medical Research and Development and Kyowa Kirin. The authors declare that there are no additional disclosures to report.

## Supporting information


**Data S1.** Supplementary Text: Quality of Life and activities of daily living measures used for study outcomes and assessments, determination of individual variables.

**Fig S1.** Caregiver disposition.
^a^As several patients were registered multiple times in the four exclusion criteria datasets, 276 patients were excluded in total.
PD, Parkinson's disease; PDQ‐8, 8‐item Parkinson's Disease Questionnaire; PDQ‐Carer, Parkinson's Disease Questionnaire‐Carer; SI, Summary Index.

**Table S1.** Baseline demographics and clinical characteristics of patients
**Table S2.** Summary of PDQ‐Carer domain scores
**Table S3.** PDQ‐Carer domain scores by H&Y stage of the patients
**Table S4.** Summary of NMSQ subdomain scores
**Table S5.** Relationship between PDQ‐Carer scores and NMSQ subdomain scores
**Table S6.** Spearman's rank correlation coefficients
