# Supplementary material for: Impact of Parkinson's Disease on Caregiver Quality of Life in Japan
Source: Mov Disord Clin Pract. 2023 Mar 14;10(4):658–63. doi: 10.1002/mdc3.13700 (PMC10105109; doi:10.1002/mdc3.13700)
Supplement: Supplementary file 1 — Data S1. Supplementary Text: Quality of Life and activities of daily living measures used for study outcomes and assessments, determination of individual variables. [file MDC3-10-658-s002.docx]

**Supplementary Text**

**Quality of Life and Activities of Daily Living Measures Used for Study Outcomes and Assessments**

The Parkinson’s Disease Questionnaire (PDQ)-Carer Summary Index (SI), a 29-item measure of health-related quality of life (HRQoL) for use in caregivers of patients with Parkinson’s disease (PD), assesses four domains of quality of life (QoL), namely social and personal activities, anxiety and depression, self-care, and strain, with the SI score being calculated to indicate the overall impact of caregiving on the QoL of caregivers. The scores for each domain of the PDQ-Carer and the SI score range from 0 to 100, with a higher score (>60 to 100) indicating worsened QoL for that particular domain or overall in the case of the SI score.

The 8-item PDQ SI (PDQ-8 SI) score provides a single index of health status that is reflective of the 39-item PDQ scores. The PDQ-8 SI rates eight specific items—mobility, activities of daily living (ADL), emotional well-being, social support, cognition, communication, bodily discomfort, and stigma—on a five-point Likert scale.

The 9-item Wearing-Off Questionnaire (WOQ-9) consists of five questions related to motor symptoms and four questions related to nonmotor symptoms.

The Nonmotor Symptoms Questionnaire (NMSQ) is a 30-item patient-based screening questionnaire designed to identify a diverse range of nonmotor symptoms grouped into nine domains—gastrointestinal tract, urinary tract, sexual function, cardiovascular, apathy/attention/memory, hallucinations/delusions, depression/anxiety/anhedonia, sleep/fatigue, and miscellaneous.

The Schwab and England Activities of Daily Living (SE-ADL) scale (0 [worst possible] to 100 [no impairment]) was used to rate the ability of patients to perform ADL.

The five-dimension, five-level version of the European Quality-of-Life Questionnaire (EQ-5D-5L), a generic instrument, was used to describe and value health across five dimensions—mobility, self-care, usual activities, pain/discomfort, and anxiety/depression.

The European Quality-of-Life Visual Analogue Scale (EQ-VAS) was used to record patients’ self-rated health on a scale from 0 (worst health you can imagine) to 100 (best health you can imagine).

**Determination of Individual Variables**

The independent variables included age, sex, and working status of the caregiver; period of care; time spent on caregiving; nursing care level and Hoehn and Yahr (H&Y) stage of the patient; frequency and types of oral PD medications taken daily; “off time” duration; duration of troublesome dyskinesias; WOQ-9, PDQ-8 SI, and SE-ADL scores; and NMSQ total score (NMSQ TS). As “off time” duration was considered the primary indicator of PD severity, it was included in the final model as a forced‑in covariate. Spearman’s correlation coefficient >0.25 was considered as a high correlation between factors (**Table S6**). SE-ADL score was removed due to a high correlation with “off time” duration. H&Y stage of the patient, nursing care level of the patient, period of care, and time spent on caregiving were highly correlated with each other. As nursing care level of the patient had the highest correlation with PDQ-Carer SI score among these four factors, H&Y stage of the patient, period of care, and time spent on caregiving were removed. The age and working status of the caregiver showed a high correlation. As working status of the caregiver had a high correlation with PDQ-Carer SI score, age of the caregiver was removed. As PDQ-8 SI had a high correlation with the nursing care level of the patient and NMSQ TS, PDQ-8 SI score was removed. Finally, “off time” duration (forced‑in covariate), sex and working status of caregiver, nursing care level of the patient, frequency and type of oral PD medications taken daily, duration of troublesome dyskinesias, WOQ-9 score, and NMSQ TS were considered independent variables.
