# Supplementary material for: Impact of Parkinson's Disease on Caregiver Quality of Life in Japan
Source: Mov Disord Clin Pract. 2023 Mar 14;10(4):658–63. doi: 10.1002/mdc3.13700 (PMC10105109; doi:10.1002/mdc3.13700)
Supplement: Supplementary file 2 — Fig S1. Caregiver disposition. aAs several patients were registered multiple times in the four exclusion criteria datasets, 276 patients were excluded in total. PD, Parkinson's disease; PDQ‐8, 8‐item Parkinson's Disease Questionnaire; PDQ‐Carer, Parkinson's Disease Questionnaire‐Carer; SI, Summary Index. [file MDC3-10-658-s001.pdf]

FIG. S1. Caregiver disposition

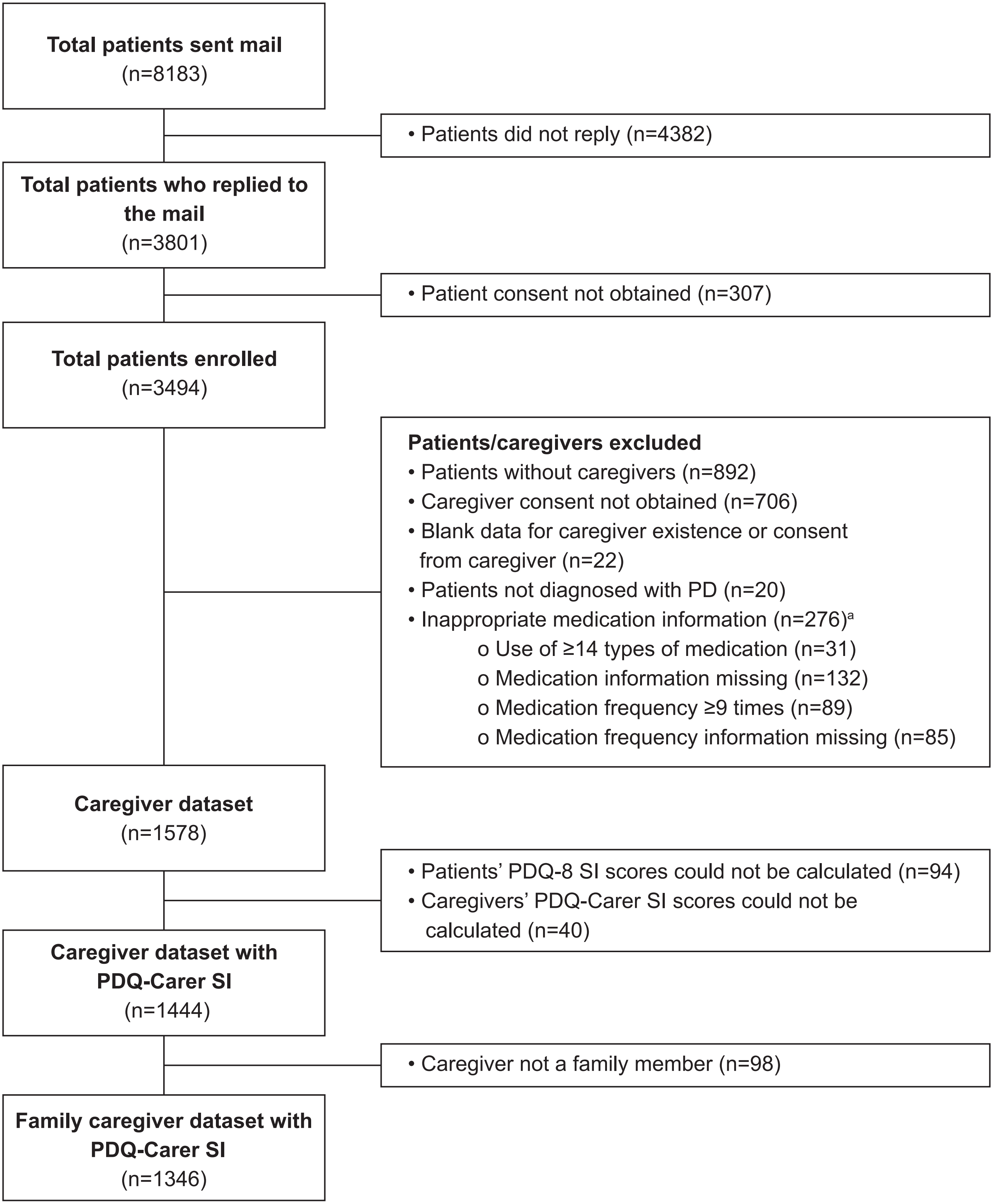

[a]As several patients were registered multiple times in the four exclusion criteria datasets, 276 patients were excluded in total.
Abbreviations: PD, Parkinson's disease; PDQ-8, 8-item Parkinson's Disease Questionnaire; PDQ-Carer, Parkinson's Disease Questionnaire–Carer; SI, Summary Index.
