# Supplementary material for: Impact of Parkinson's Disease on Caregiver Quality of Life in Japan
Source: Mov Disord Clin Pract. 2023 Mar 14;10(4):658–63. doi: 10.1002/mdc3.13700 (PMC10105109; doi:10.1002/mdc3.13700)
Supplement: Supplementary file 3 — Table S1. Baseline demographics and clinical characteristics of patients Table S2. Summary of PDQ‐Carer domain scores Table S3. PDQ‐Carer domain scores by H&Y stage of the patients Table S4. Summary of NMSQ subdomain scores Table S5. Relationship between PDQ‐Carer scores and NMSQ subdomain scores Table S6. Spearman's rank correlation coefficients [file MDC3-10-658-s003.docx]

**TABLE S1.** Baseline demographics and clinical characteristics of patients

| **Demographic and clinical characteristics** | **N=1346**  **n (%)** | **Mean (SD)** |
| --- | --- | --- |
| Demographics |  |  |
| Age, years | 1342 | 71.8 (7.6) |
| <65 | 195 (14.5) |  |
| ≥65 | 1147 (85.2) |  |
| Sex, female | 636 (47.3) |  |
| Medical history |  |  |
| Age at PD diagnosis, years | 1312 | 59.6 (10.1) |
| Duration of PD, years | 1311 | 10.9 (6.7) |
| H&Y stage |  |  |
| 1 | 21 (1.6) |  |
| 2 | 105 (7.8) |  |
| 3 | 541 (40.2) |  |
| 4 | 280 (20.8) |  |
| 5 | 57 (4.2) |  |
| Working status |  |  |
| Full-time | 32 (2.4) |  |
| Part-time | 19 (1.4) |  |
| Other (eg, on leave) | 30 (2.2) |  |
| Student | 1 (0.1) |  |
| Housewife/househusband | 430 (31.9) |  |
| Seeking a job/unemployed | 23 (1.7) |  |
| Retired | 539 (40.0) |  |
| Others | 259 (19.2) |  |
| PD treatment |  |  |
| Use of oral medication |  |  |
| Frequency of oral PD medications a day | 1346 | 4.3 (1.6) |
| Frequency of oral levodopa medications a day | 1119 | 3.9 (1.6) |
| Types of oral PD medications taken daily | 1346 | 4.7 (2.6) |
| Use of device-aided therapy |  |  |
| DBS | 122 (9.1) |  |
| LCIG | 13 (1.0) |  |
| PD symptoms |  |  |
| Duration of off time, hours/day | 1213 | 2.16 (2.45) |
| Duration of troublesome dyskinesia, hours/day | 1241 | 0.82 (1.69) |
| Presence of WO as per WOQ-9 | 715 (66.2) |  |
| SE-ADL score | 1267 | 59.5 (19.9) |
| PDQ-8 SI score | 1346 | 39.9 (20.0) |
| EQ-5D-5L SI score | 1296 | 0.489 (0.194) |
| EQ-VAS score | 1298 | 56.4 (18.9) |
| NMSQ TS | 1052 | 16.1 (5.5) |
| Unknown/missing data are not listed.  Abbreviations: DBS, deep brain stimulation; EQ-5D-5L SI, five-dimension, five-level version of the European Quality-of-Life Questionnaire Summary Index; EQ-VAS, European Quality-of-Life Visual Analogue Scale; H&Y, Hoehn and Yahr; LCIG, levodopa-carbidopa intestinal gel; NMSQ TS, Nonmotor Symptoms Questionnaire total score; PD, Parkinson’s disease; PDQ-8, 8-item Parkinson’s Disease Questionnaire; SD, standard deviation; SE-ADL, Schwab and England Activities of Daily Living; SI, Summary Index; WO, wearing-off; WOQ-9, 9-item Wearing-Off Questionnaire. | | |

**TABLE S2.** Summary of PDQ-Carer domain scores

| **PDQ-Carer** | **n (N=1346)** | **Mean (SD)** | **Median (min, max)** |
| --- | --- | --- | --- |
| Personal and social activities | 1255 | 38.0 (26.5) | 33.3 (0, 100) |
| Anxiety and depression | 1320 | 45.5 (25.8) | 41.7 (0, 100) |
| Self-care | 1306 | 32.5 (24.4) | 30.0 (0, 100) |
| Strain | 1286 | 40.8 (23.9) | 37.5 (0, 100) |
| Summary index | 1346 | 39.3 (23.8) | 35.3 (0, 100) |
| Abbreviations: max, maximum; min, minimum; PDQ-Carer, Parkinson’s Disease Questionnaire–Carer; SD, standard deviation. | | | |

**TABLE S3.** PDQ-Carer domain scores by H&Y stage of the patients

| **PDQ-Carer**  **domain** | **H&Y stage** | | | | | | | | | | | | | | | **p-value** |
| --- | --- | --- | --- | --- | --- | --- | --- | --- | --- | --- | --- | --- | --- | --- | --- | --- |
|  | **1** | | | **2** | | | **3** | | | **4** | | | **5** | | |  |
|  | **n** | **Mean (SD)** | **Median (min, max)** | **n** | **Mean (SD)** | **Median (min, max)** | **n** | **Mean (SD)** | **Median (min, max)** | **n** | **Mean (SD)** | **Median (min, max)** | **n** | **Mean (SD)** | **Median (min, max)** |  |
| Personal and social activities | 20 | 27.8 (25.6) | 26.0  (0, 100) | 96 | 24.5 (20.8) | 18.8  (0, 85) | 513 | 33.1 (24.3) | 27.1  (0, 100) | 260 | 47.3 (25.9) | 45.8  (0, 100) | 55 | 64.8 (23.4) | 66.7  (0, 100) | <0.0001 |
| Anxiety and  depression | 20 | 39.0 (30.3) | 31.3  (0, 100) | 104 | 35.4 (21.8) | 33.3  (0, 96) | 533 | 42.4 (25.0) | 37.5  (0, 100) | 272 | 51.1 (25.6) | 50.0  (0, 100) | 57 | 61.7 (25.1) | 66.7  (0, 100) | <0.0001 |
| Self-care | 20 | 28.3 (27.8) | 20.0  (0, 100) | 100 | 20.6 (17.9) | 20.0  (0, 75) | 525 | 28.8 (22.7) | 25.0  (0, 100) | 272 | 39.6 (24.3) | 35.0  (0, 100) | 56 | 54.6 (24.9) | 55.0  (0, 100) | <0.0001 |
| Strain | 21 | 32.3 (29.1) | 20.8  (0, 100) | 100 | 32.7 (20.0) | 29.2  (0, 83) | 517 | 37.8 (22.9) | 33.3  (0, 100) | 270 | 47.7 (23.3) | 45.8  (0, 100) | 55 | 56.4 (24.9) | 54.2  (0, 100) | <0.0001 |
| Summary  index | 21 | 30.5 (26.6) | 25.0  (1, 100) | 105 | 28.6 (19.1) | 25.0  (2, 81) | 541 | 35.4 (22.2) | 31.0  (0, 100) | 280 | 46.5 (23.5) | 44.8  (1, 100) | 57 | 60.5 (22.3) | 64.7  (0, 97) | <0.0001 |
| Abbreviations: H&Y, Hoehn and Yahr; max, maximum; min, minimum; PDQ-Carer, Parkinson’s Disease Questionnaire–Carer; SD, standard deviation. | | | | | | | | | | | | | | | | |

| **NMSQ subdomain** | **Number of questions** | **n (N=1346)** | **Mean (SD)** | **Median (min, max)** |
| --- | --- | --- | --- | --- |
| Digestive | 7 | 1268 | 3.4 (1.7) | 3.0 (0, 7) |
| Urinary | 2 | 1318 | 1.6 (0.6) | 2.0 (0, 2) |
| Memory | 3 | 1314 | 2.1 (1.0) | 2.0 (0, 3) |
| Autonomic | 2 | 1303 | 1.1 (0.7) | 1.0 (0, 2) |
| Sleep disorder | 5 | 1237 | 2.8 (1.4) | 3.0 (0, 5) |
| Perceptual | 2 | 1305 | 0.9 (0.9) | 1.0 (0, 2) |
| Mood | 2 | 1305 | 1.0 (0.9) | 1.0 (0, 2) |
| Sexual function | 2 | 1253 | 0.5 (0.8) | 0.0 (0, 2) |
| Others | 5 | 1275 | 2.7 (1.3) | 3.0 (0, 5) |
| Total score | 30 | 1052 | 16.1 (5.5) | 17.0 (0, 30) |
| Abbreviations: max, maximum; min, minimum; NMSQ, Nonmotor Symptoms Questionnaire; SD, standard deviation. | | | | |

**TABLE S4.** Summary of NMSQ subdomain scores

**TABLE S5.** Relationship between PDQ-Carer scores and NMSQ subdomain scores

|  |  | **Spearman's rank correlation coefficient** | | | | | | | | | |
| --- | --- | --- | --- | --- | --- | --- | --- | --- | --- | --- | --- |
| **PDQ-Carer**  **domain** |  | **NMSQ subdomain** | | | | | | | | | **NMSQ-TS** |
|  |  | **Digestive** | **Urinary** | **Memory** | **Autonomic** | **Sleep disorders** | **Perceptual** | **Mood** | **Sexual function** | **Miscellaneous** |  |
| Personal and social activities |  | 0.2010 | 0.1265 | 0.2815 | 0.1205 | 0.2092 | 0.3281 | 0.1708 | 0.1166 | 0.1376 | 0.3252 |
|  | p-value | <0.0001 | <0.0001 | <0.0001 | <0.0001 | <0.0001 | <0.0001 | <0.0001 | <0.0001 | <0.0001 | <0.0001 |
| Anxiety and  depression |  | 0.1881 | 0.1339 | 0.2465 | 0.1176 | 0.1911 | 0.2764 | 0.1663 | 0.1298 | 0.1688 | 0.3025 |
|  | p-value | <0.0001 | <0.0001 | <0.0001 | <0.0001 | <0.0001 | <0.0001 | <0.0001 | <0.0001 | <0.0001 | <0.0001 |
| Self-care |  | 0.2171 | 0.1144 | 0.2531 | 0.1266 | 0.2111 | 0.2916 | 0.1822 | 0.1110 | 0.1787 | 0.3382 |
|  | p-value | <0.0001 | <0.0001 | <0.0001 | <0.0001 | <0.0001 | <0.0001 | <0.0001 | 0.0001 | <0.0001 | <0.0001 |
| Strain |  | 0.1700 | 0.1136 | 0.2506 | 0.1078 | 0.2078 | 0.3123 | 0.1938 | 0.1333 | 0.1362 | 0.3053 |
|  | p-value | <0.0001 | <0.0001 | <0.0001 | 0.0001 | <0.0001 | <0.0001 | <0.0001 | <0.0001 | <0.0001 | <0.0001 |
| Summary  index |  | 0.2103 | 0.1319 | 0.2829 | 0.1259 | 0.2194 | 0.3309 | 0.1861 | 0.1295 | 0.1582 | 0.3400 |
|  | p-value | <0.0001 | <0.0001 | <0.0001 | <0.0001 | <0.0001 | <0.0001 | <0.0001 | <0.0001 | <0.0001 | <0.0001 |
| Abbreviations: NMSQ-TS, Nonmotor Symptoms Questionnaire total score; PDQ-Carer, Parkinson’s Disease Questionnaire–Carer. | | | | | | | | | | | |

**TABLE S6.** Spearman's rank correlation coefficients

|  | **H&Y stage of the patient** | **Types of oral PD medications taken daily** | **Frequency and types of oral PD medications taken daily** | **“Off time” duration** | **Duration of troublesome dyskinesias** | **Age of caregiver** | **Nursing care level** | **Period of care** | **Time spent on caregiving** | **Sex of caregiver** | **Working status of caregiver** | **WOQ-9** | **SE-ADL** | **NMSQ TS** | **PDQ-Carer SI** | **PDQ-8 SI** |
| --- | --- | --- | --- | --- | --- | --- | --- | --- | --- | --- | --- | --- | --- | --- | --- | --- |
| H&Y stage of the patient |  | <0.0001 | 0.0002 | <0.0001 | 0.0352 | 0.2096 | <0.0001 | <0.0001 | <0.0001 | 0.7022 | 0.4331 | 0.0372 | <0.0001 | <0.0001 | <0.0001 | <0.0001 |
| Types of oral PD medications taken daily |  |  | <0.0001 | 0.0547 | 0.0072 | 0.9111 | <0.0001 | <0.0001 | 0.0207 | 0.8824 | 0.9423 | 0.0223 | <0.0001 | <0.0001 | 0.0003 | 0.0001 |
| Frequency and types of oral PD medications taken daily |  |  |  | <0.0001 | <0.0001 | 0.0443 | <0.0001 | <0.0001 | 0.114 | 0.0926 | 0.576 | <0.0001 | 0.0001 | <0.0001 | 0.054 | 0.0557 |
| “Off time” duration |  |  |  |  | <0.0001 | 0.0007 | <0.0001 | <0.0001 | 0.2922 | 0.1193 | 0.1221 | <0.0001 | <0.0001 | <0.0001 | <0.0001 | <0.0001 |
| Duration of troublesome dyskinesias |  |  |  |  |  | 0.4734 | 0.0011 | 0.003 | 0.3727 | 0.0157 | 0.518 | 0.001 | <0.0001 | 0.0002 | 0.0095 | <0.0001 |
| Age of caregiver |  |  |  |  |  |  | 0.2575 | 0.0003 | <0.0001 | <0.0001 | <0.0001 | 0.0027 | 0.0193 | 0.302 | 0.0043 | 0.6879 |
| Nursing care level |  |  |  |  |  |  |  | <0.0001 | <0.0001 | 0.0484 | 0.0018 | 0.3599 | <0.0001 | <0.0001 | <0.0001 | <0.0001 |
| Period of care |  |  |  |  |  |  |  |  | <0.0001 | 0.056 | <0.0001 | 0.0004 | <0.0001 | <0.0001 | <0.0001 | <0.0001 |
| Time spent on caregiving |  |  |  |  |  |  |  |  |  | 0.1866 | <0.0001 | 0.1203 | <0.0001 | <0.0001 | <0.0001 | <0.0001 |
| Sex of caregiver |  |  |  |  |  |  |  |  |  |  | 0.6121 | 0.2987 | 0.93 | 0.1084 | <0.0001 | 0.5616 |
| Working status of caregiver |  |  |  |  |  |  |  |  |  |  |  | 0.0067 | 0.0002 | 0.046 | <0.0001 | 0.809 |
| WOQ-9 |  |  |  |  |  |  |  |  |  |  |  |  | 0.7628 | <0.0001 | 0.3522 | 0.6058 |
| SE-ADL |  |  |  |  |  |  |  |  |  |  |  |  |  | <0.0001 | <0.0001 | <0.0001 |
| NMSQ TS |  |  |  |  |  |  |  |  |  |  |  |  |  |  | <0.0001 | <0.0001 |
| PDQ-Carer SI |  |  |  |  |  |  |  |  |  |  |  |  |  |  |  | <0.0001 |
| PDQ-8 SI |  |  |  |  |  |  |  |  |  |  |  |  |  |  |  |  |
| Abbreviations: H&Y, Hoehn and Yahr; NMSQ TS, Nonmotor Symptoms Questionnaire total score; PD, Parkinson’s disease; PDQ-8, 8-item Parkinson’s Disease Questionnaire; PDQ-Carer, PDQ-Carer, Parkinson’s Disease Questionnaire–Carer; SE-ADL, Schwab and England Activities of Daily Living; SI, Summary Index; WOQ-9, 9-item Wearing-Off Questionnaire. | | | | | | | | | | | | | | | | |
